# Myd88 Is Required for an Antibody Response to Retroviral Infection

**DOI:** 10.1371/journal.ppat.1000298

**Published:** 2009-02-13

**Authors:** Edward P. Browne, Dan R. Littman

**Affiliations:** 1 The Kimmel Center for Biology and Medicine at the Skirball Institute, New York University School of Medicine, New York, New York, United States of America; 2 Departments of Pathology and Microbiology, New York University School of Medicine, New York, New York, United States of America; 3 Howard Hughes Medical Institute, New York University School of Medicine, New York, New York, United States of America; University of Geneva, Switzerland

## Abstract

Although retroviruses have been extensively studied for many years, basic questions about how retroviral infections are detected by the immune system and which innate pathways are required for the generation of immune responses remain unanswered. Defining these pathways and how they contribute to the anti-retroviral immune responses would assist in the development of more effective vaccines for retroviral pathogens such as HIV. We have investigated the roles played by CD11c^+^ dendritic cells (DCs) and by Toll-like receptor (TLR) signaling pathways in the generation of an anti-retroviral immune response against a mouse retroviral pathogen, Friend murine leukemia virus (F-MLV). Specific deletion of DCs during F-MLV infection caused a significant increase in viral titers at 14 days post-infection, indicating the importance of DCs in immune control of the infection. Similarly, Myd88 knockout mice failed to control F-MLV, and sustained high viral titers (10^7^ foci/spleen) for several months after infection. Strikingly, both DC-depleted mice and Myd88 knockout mice exhibited only a partial reduction of CD8^+^ T cell responses, while the IgG antibody response to F-MLV was completely lost. Furthermore, passive transfer of immune serum from wild-type mice to Myd88 knockout mice rescued control of F-MLV. These results identify TLR signaling and CD11c^+^ DCs as playing critical roles in the humoral response to retroviruses.

## Author Summary

Efforts to develop vaccines against the retrovirus HIV by inducing immune responses involving antibodies or T cells have been unsuccessful. Although antibodies can be generated against HIV, they fail to neutralize the virus. Thus, a more fundamental understanding of how neutralizing antibody responses to retroviral pathogens are generated is required. We have used a mouse retrovirus to demonstrate that Myd88, a molecule centrally involved in innate immune system signaling, is required to generate an antibody response during retroviral infection. Myd88 also contributed to, but was not strictly required for, the T cell response. Myd88 is known to participate in a signaling pathway that activates inflammation in response to microbial molecules. Understanding how this pathway contributes to anti-retroviral antibody responses may be useful for the development of a vaccine that can effectively block HIV.

## Introduction

The HIV pandemic has spurred intensive research into retroviruses, and yet an effective vaccine for HIV has remained elusive. Acute HIV infection stimulates both B and T cell responses, but the antibody response is ineffective, possibly due to shielding of neutralizing epitopes [1],[2]. By contrast, HIV-specific CD8^+^ T cells are able to control infection early on, but become progressively less effective during the chronic phase of infection due to mechanisms that remain unclear [3],[4]. Vaccines designed to stimulate protective B cell or T cell responses have been used in clinical trials, but have been unsuccessful at either preventing infection or reducing viral titers in infected individuals [5]. Thus, a more fundamental understanding of anti-retroviral immune responses is needed to develop an effective vaccine. Basic questions that have not been answered include: 1) Which antigen presenting cell populations are necessary or sufficient to generate an immune response? 2) Which innate signaling pathways detect retroviral infection *in vivo* and are responsible for initiating adaptive immune responses?

There have been major advances during the past decade in our understanding of how the innate immune system functions to limit viral growth and stimulate T and B cell- dependent adaptive immune responses. It is now understood that microbial products that serve as pathogen-associated molecular patterns (PAMPs) are detected by germline-encoded innate immune receptors, such as the members of the Toll-like receptor (TLR) family [6]. These receptors are prominently expressed in antigen-presenting cells such as dendritic cells (DCs) that function at the interface between innate and adaptive immunity. Humans encode at least ten TLRs while mice encode at least twelve. Products of bacterial metabolic pathways are recognized by specific TLRs such as LPS by TLR4 and flagellin by TLR5 [7]. Viruses, by contrast, are thought to be detected by mechanisms that involve endosomal localization of viral nucleic acids. ssRNA is detected by TLR7 [8],[9], dsRNA is detected by TLR3 [10], and CpG dsDNA is recognized by TLR9 [11]. All TLRs except for TLR3 signal through a pathway that involves the adaptor Myd88 [12]. Upon stimulation, Myd88 is recruited to the TLR as a dimer, and activates the kinases IRAK1 and IRAK4. This activates a signaling cascade that ultimately leads to the activation of the pro-inflammatory transcription factor NF-κB, as well as the MAP kinase and JNK pathways [13]. In the absence of Myd88, TLR3 and TLR4 are able to signal through another adaptor, TRIF [14].

Friend murine leukemia virus (F-MLV) is a complex gamma-retrovirus that has been used to understand basic principles of anti-retroviral immune responses and mechanisms of chronic infection [15]. It consists of a replication-competent helper virus and a replication-defective spleen focus-forming virus (SFFV). SFFV encodes a protein, gp55, which binds to the erythropoietin receptor and causes hyper-proliferation of erythroid precursors and splenomegaly. The susceptibility of mice to F-MLV is affected by a number of host genes. In mouse strains such as Balb/c, immune responses are ineffective and sustained high virus titers eventually lead to erythroid leukemia. In C57BL/6 mice, acute infection is controlled by CD8^+^ T cells and neutralizing antibodies, but the virus is not completely cleared and establishes a low level persistent infection [16].

Retroviral particles for HIV and F-MLV possess potential TLR ligands, and could conceivably be detected by TLR signaling [17]. Indeed, some evidence has indicated that plasmacytoid dendritic cells secrete type I interferons in response to HIV by way of a TLR7-dependent pathway [18]. However, the role played by TLRs in generating an anti-retroviral immune response *in vivo* is unknown.

In this study, we addressed the roles played by CDllc^+^ DCs and Myd88-dependent signaling in the immune response to F-MLV infection *in vivo*. Using mice that permit specific deletion of CD11c^+^ DCs as well as Myd88-deficient mice, we determined that DCs and Myd88 contribute to, but are not required for, T cell responses to F-MLV, but are absolutely required for the generation of virus-neutralizing antibodies.

## Results

### F-MLV replication kinetics *in vivo*

**Figure 1:**
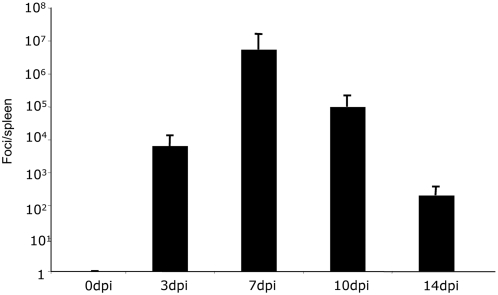
F-MLV growth curve *in vivo*. C57BL/6 mice were infected with F-MLV. At the time indicated, the number of viral foci in the spleen was measured by focus-forming assay on *Mus Dunni* cells. Each bar represents the average of five mice. The data shown are representative of two independent experiments.

Previous reports demonstrated that F-MLV titers in the spleens of infected mice peak at 14 days post-infection (dpi) before they subside to a low level during the phase of chronic infection. However, F-MLV stocks used in the earlier studies were contaminated with Lactate Dehydrogenase-Elevating Virus (LDV), which has been shown to delay the immune response to F-MLV [19]. To establish growth kinetics of F-MLV *in vivo* in the absence of LDV, we infected C57BL/6 mice with F-MLV and measured viral titers in the spleen at different times post-infection. The number of foci per spleen peaked around 7 dpi at approximately 10^6^ foci per spleen, and by 14 dpi the viral titer had been reduced to between 10^2^ and 10^3^ foci per spleen (Figure 1). This growth curve is consistent with a recent report using LDV-free F-MLV [19].

### F-MLV infection causes dendritic cell maturation *in vivo*

**Figure 2:**
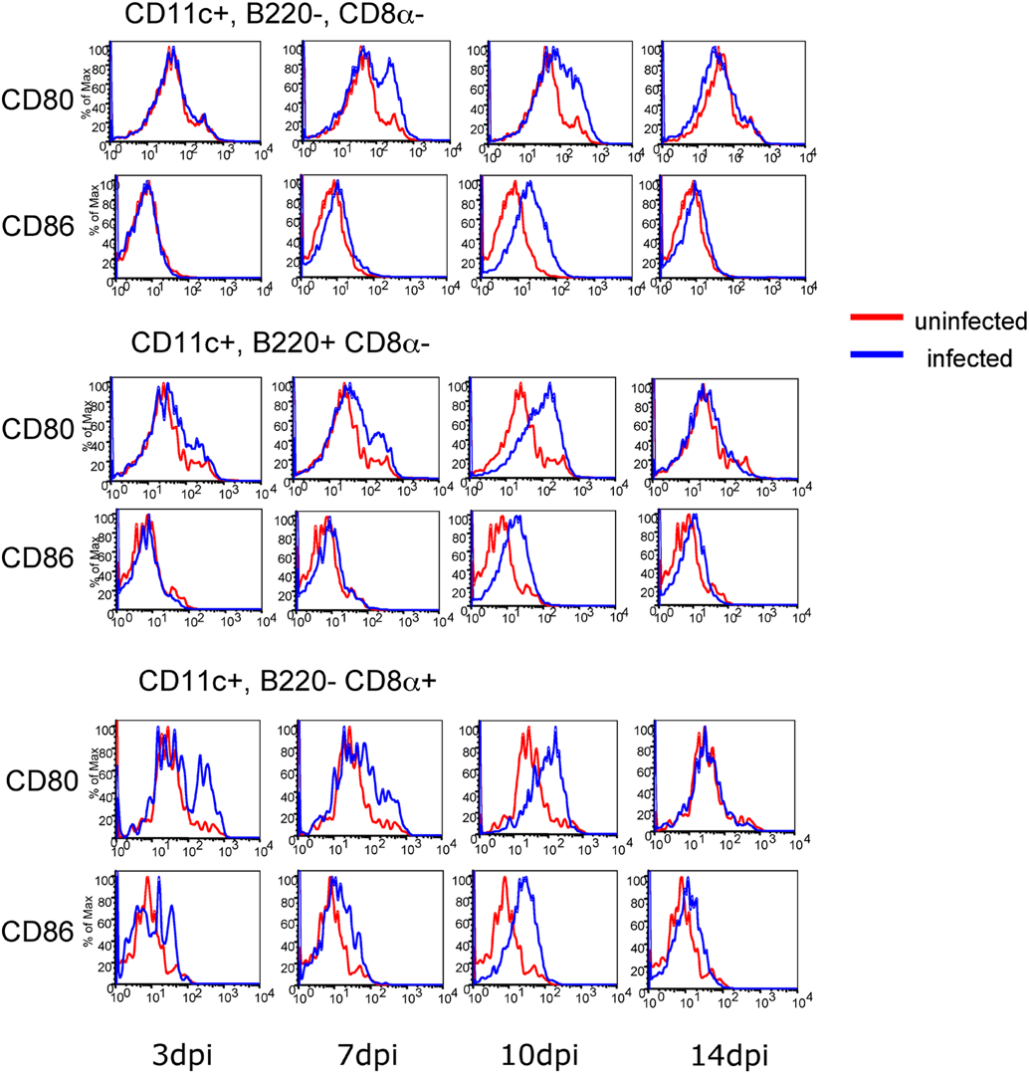
F-MLV infection causes dendritic cell maturation in vivo. C57BL/6 mice were infected with F-MLV. At the times indicated, dendritic cell populations in the spleen were analyzed for maturation by staining for CD80 and CD86. Each FACS plot is representative of five mice. Data from uninfected mice are shown in red, while infected mice are shown in blue. The data shown are representative of three independent experiments.

Dendritic cells (DCs) are specialized cells that carry out a number of important roles. They detect pathogens through innate receptors and activate signaling pathways that lead to the expression of cytokines. Furthermore, they present foreign antigens to T cells by way of MHCI and MHCII molecules. Mouse splenic DCs consist of three major subsets with specialized functions. CDllc^+^ CD8α^−^ B220^−^ (“myeloid”) DCs are the classical antigen-presenting cell and are important for activation of CD4^+^ T cells. CDllc^Int^ B220^+^ CD8α^−^ (“plasmacytoid”) DCs are potent producers of type 1 interferons [20]. CDllc^+^ B220^−^ CD8α^+^ DCs are specialized for cross presentation and are important for activating CD8^+^ T cells [21]. When DCs detect foreign pathogens they mature and up-regulate their antigen presenting machinery as well as costimulatory molecules such as CD80 and CD86. To determine if F-MLV causes activation of specific DC subsets during infection, we infected C57BL/6 mice with F-MLV and examined the expression of CD80 and CD86 on all three splenic DC subsets at different times post-infection. CD80 and CD86 were upregulated on all three DC subsets beginning at 7 dpi (Figure 2). DC expression of CD80 and CD86 levels returned to baseline by 14 dpi. This indicates that all three DC subsets mature in response to F-MLV infection *in vivo*, and that DC activation peaks between 7–10 dpi.

### DCs are required for control of F-MLV replication

Immune control of F-MLV requires both CD8^+^ cytotoxic T lymphocyte (CTL) and neutralizing antibody responses [22]. Several cell types, including DCs, macrophages and B cells, are capable of antigen presentation to T cells, but for most pathogens it is unclear which of these cell types is explicitly required for the generation of specific immune responses. To investigate the role of CD11c^+^ DCs in immune function, we previously developed transgenic mice that express the diphtheria toxin receptor (DTR) under the control of the CD11c promoter [23]. Since expression of DTR renders murine cells susceptible to killing by diphtheria toxin (DT), this transgene allows for specific deletion of CDllc-expressing DCs *in vivo* by the administration of DT. Because repeated administration of DT to these mice is lethal within 7 days due to an unknown mechanism, we generated bone marrow chimeras, by lethally irradiating C57BL/6 mice and reconstituting them with wild-type or CDllc-DTR transgenic bone marrow. Mice receiving the transgenic bone marrow are viable through at least 2 weeks of repeated DT injection.

**Figure 3:**
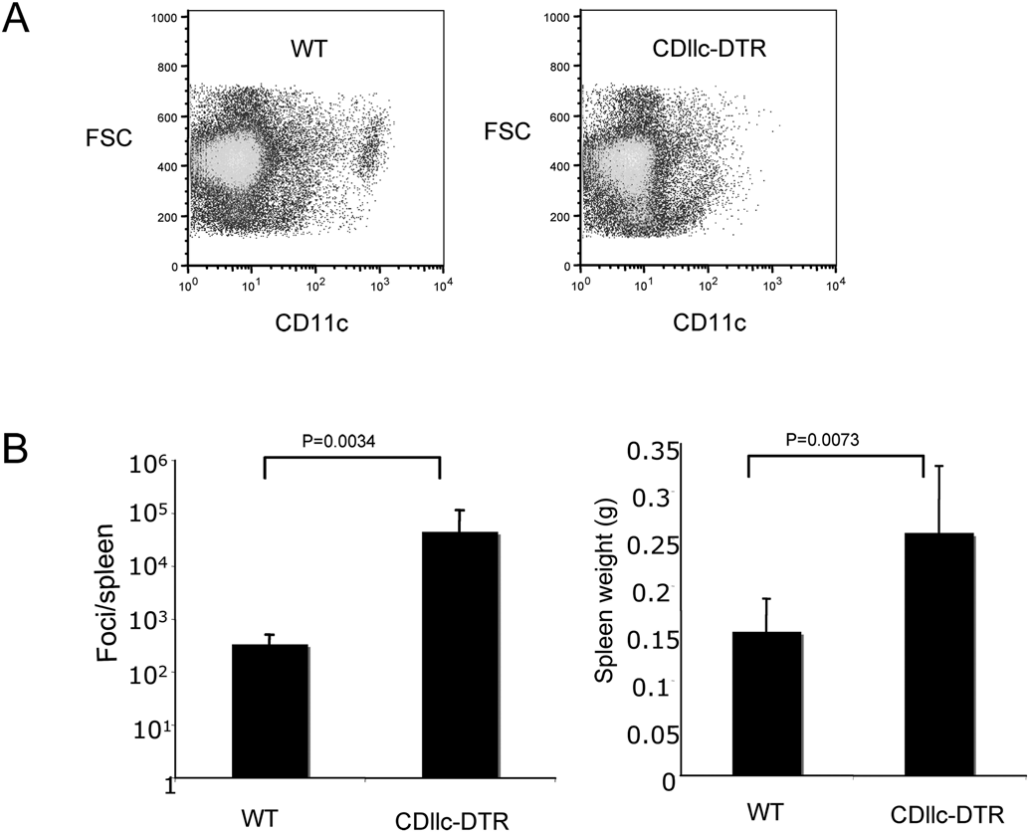
Dendritic cells are required for immune control of F-MLV infection. Lethally irradiated C57BL/6 mice that had been reconstituted with wild-type (WT) or CD11c-DTR bone marrow were infected intravenously with F-MLV. DT was injected intraperitoneally into both wild-type and CD11c-DTR mice every day from one day prior to infection until 14 days post-infection. At 14 days post-infection, spleens were harvested, the level of DC depletion analyzed (A), and the number of viral foci per spleen and spleen weight were measured (B). Each bar is the average of seven mice for each group. The data shown are representative of three independent experiments.

To investigate the role played by CD11c^+^ DCs in the immune response to a retrovirus, we infected the chimeric mice with F-MLV. To delete DCs, we injected 200 ng DT daily from one day prior to infection until 14 dpi. At 14 dpi, the mice were sacrificed, and the extent of DC depletion in the spleen was determined by flow cytometry. Deletion of CD11c^+^ cells was typically around 90% (Figure 3A). Strikingly, the number of infectious foci in the spleens of DC-depleted mice was 100-fold higher than in non-depleted mice (Figure 3B, left panel). This indicates that CDllc^+^ DCs play an essential role in immune control of F-MLV, and that other antigen presenting cells are not sufficient for full activation of anti-retroviral immune responses. DC-depleted mice also exhibited increased splenomegaly (Figure 3B, right panel), indicative of higher levels of virus.

To learn about the temporal requirement for DCs in immune control of F-MLV infection, we also depleted DCs from day 4 to 14 following infection. We had previously observed strong activation of DC populations in the spleen at 7–10 dpi (Figure 2). Interestingly, depletion from day 4–14 post-infection did not result in a significant increase in virus titers in the spleen relative to undepleted mice (not shown). This suggests that the presence of DCs during the first four days of infection is sufficient to establish immune control, and that the mature DC populations visible later in infection are not required.

### DCs are required for the neutralizing antibody response to F-MLV

**Figure 4:**
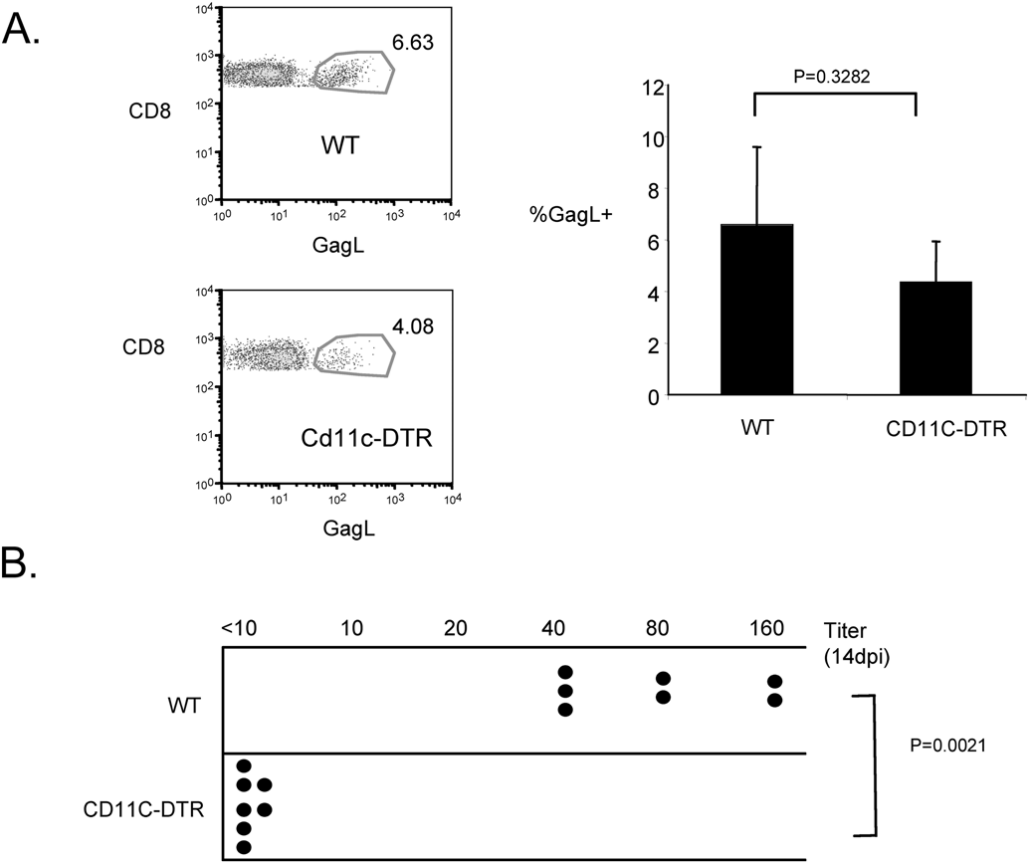
Dendritic cells are required for the neutralizing antibody response to F-MLV. (A) In dendritic cell-depleted F-MLV–infected mice, the CD8^+^ T cell response to F-MLV at 14 dpi was measured by staining splenocytes with the D^b^-GagL tetramer. Representative FACS plots of D^b^GagL staining gated on CD8^+^ cells are shown on the left. The proportion of CD8^+^ T cells that stained with GagL was calculated for each sample and is shown on the right. Each bar represents the average value of four mice. (B) In dendritic cell-depleted F-MLV–infected mice, the titer of F-MLV–neutralizing antibodies in the serum was measured. Numerical values represent the maximum dilution of serum that neutralized F-MLV infection of *Mus Dunni* cells. Each dot represents an individual mouse. The data shown are representative of three independent experiments.

Since DC-depleted mice exhibited reduced ability to control F-MLV, we wished to determine whether either the CTL or neutralizing antibody response was affected by the depletion. To measure the role of CD11c^+^ DCs in the CTL response, we infected chimeras reconstituted with wild-type or CDllc-DTR bone marrow with F-MLV. DCs were depleted by injection of DT from one day prior to infection until 14 days post-infection. At 14 dpi, splenocytes were stained with the D^b^-GagL tetramer. This tetramer typically stains 5–10% of CD8^+^ CTLs in a F-MLV–infected wild-type mouse, but does not stain CD8^+^ T cells from naïve mice [16]. We found that the proportion of CD8^+^ T cells that stained with D^b^-GagL was only slightly reduced in DC-depleted mice, suggesting that high levels of CD11c^+^ DCs are not required for the generation of CTL responses to F-MLV (Figure 4A). It is possible that only a small number of DCs is sufficient for activating a CD8^+^ T cell responses, or that a non-depleted APC population mediates CD8^+^ T cell activation.

To assess the role of DCs in the generation of neutralizing antibodies against F-MLV, we infected the bone marrow chimeras with F-MLV and depleted DCs by injection of DT from day −1 to day 14 post-infection. At 14 dpi, we harvested peripheral blood from infected mice and isolated serum. We then measured the titer of F-MLV–specific neutralizing antibodies in the serum by determining the maximum dilution of serum that was still sufficient to neutralize F-MLV infection of *Mus Dunni* cells in tissue culture. In wild-type infected mice, neutralizing antibody titers were typically between 40–160. Strikingly, in DC depleted mice, neutralizing antibodies were undetectable, with titers<10 (Figure 4B). These results demonstrate that CD11c^+^ DCs play an essential role in the generation of F-MLV–specific neutralizing antibodies, but are less important for CTL responses.

### Myd88 is required for immune control of F-MLV

**Figure 5:**
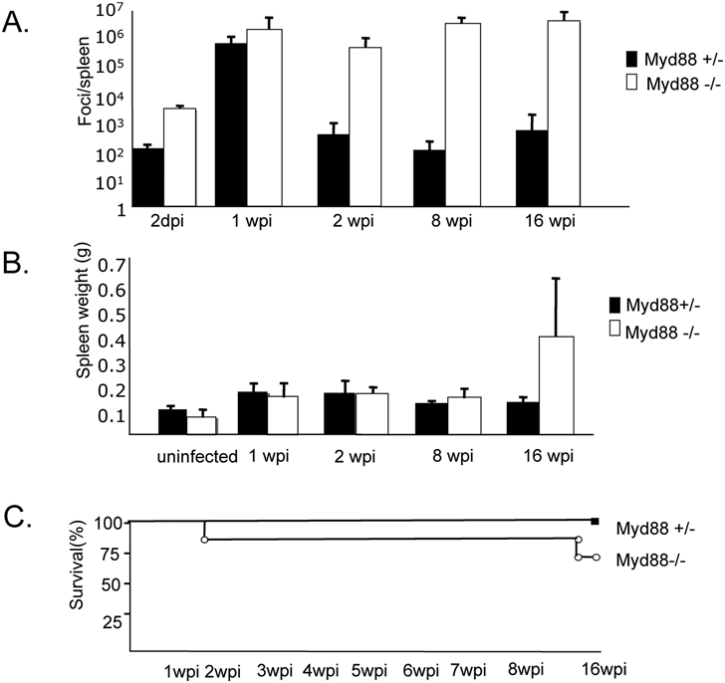
Myd88 is required for immune control of F-MLV. Myd88 knockout mice and heterozygous littermates were infected with F-MLV. (A) At the times indicated, the viral titer in the spleen was measured. Each bar is the average of 4–8 mice. (B) At the times indicated, the weight of the spleen was measured. Each bar is the average of 4–8 mice. (C) The survival of Myd88 knockout mice and heterozygous littermates was measured over a 16-week period. The data shown are representative of two independent experiments.

Because F-MLV particles contain molecules such as ssRNA that could activate TLR signaling, we reasoned that infection might be detected *via* a TLR-dependent pathway. To test whether TLR signaling was required for immune control of F-MLV, we infected mice heterozygous or homozygous for a null Myd88 allele and analyzed virus levels in the spleens at various time-points post-infection. We also compared wild-type mice to heterozygous mice at several time-points over an eight week period and found no significant difference in viral titers, so all comparisons in subsequent studies were between heterozygous and homozygous mutant mice. In the first 7 days after infection, viral levels were slightly higher in Myd88 null mice than in heterozygotes, but they reached a similar peak level at one week post-infection (wpi). At two wpi, however, viral titers were dramatically higher in Myd88 knockout mice. In heterozygous mice, titers dropped to between 10^2^ and 10^3^ foci per spleen, whereas in Myd88 knockout mice virus titers remained at between 10^5^ and10^6^ foci per spleen. Furthermore, at 8 wpi and 16 wpi, heterozygous mice had developed a low level chronic infection at 10^2^–10^3^ foci per spleen, while Myd88 knockout mice exhibited high (10^6^–10^7^ foci per spleen) levels of virus infection (Figure 5A). This suggested that some aspect of immune control of F-MLV is impaired in the absence of Myd88. In contrast with DC depletion, splenomegaly was not enhanced relative to that in heterozygous mice at 2 wpi. By 16 wpi, however, some Myd88 knockout mice exhibited significantly enlarged spleens (Figure 5B). Interestingly, Myd88 knockout mice did not show a significant increase in mortality relative to heterozygous mice over 16 weeks of infection, and infected knockout mice seemed otherwise healthy despite maintaining high virus titers (Figure 5C).

### DC maturation *in vivo* partially requires Myd88

**Figure 6:**
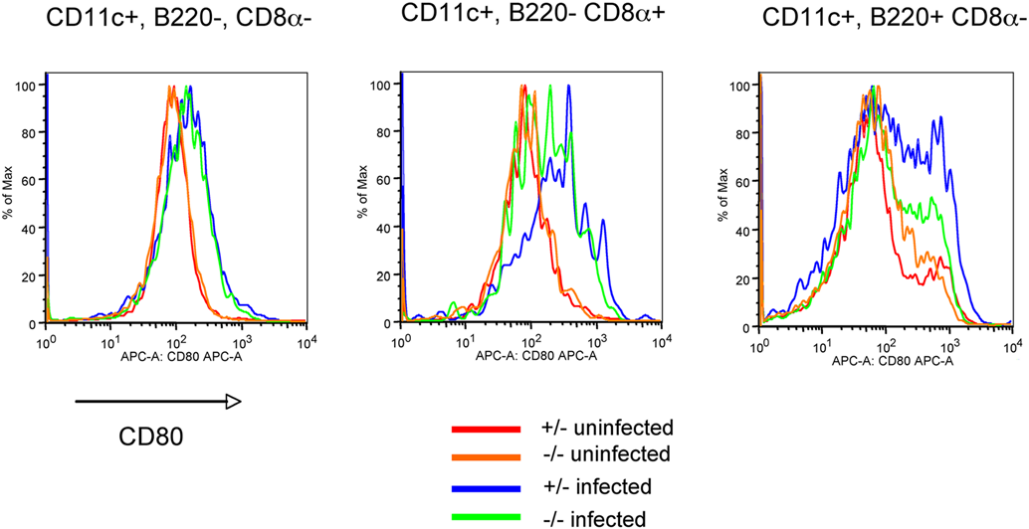
Dendritic cell maturation during F-MLV infection is partially dependent on Myd88. Myd88 knockout mice and heterozygous littermates were infected with F-MLV. At 7 dpi, splenic DCs were stained for expression of CD80. The populations were gated as follows: CD11c^+^ B220^−^ CD8α^−^, CD11c^+^ B220- CD8α^+^, and CD11c^int^, B220^+^ CD8α^+^. A representative histogram of CD80 staining from three independent experiments using 4–6 mice is shown for each population.

We had previously observed that the three major DC subsets in the spleen undergo maturation between 7 and 10 days post-infection. To determine if DC maturation was dependent on Myd88, we analyzed expression of CD80 at 7 dpi on splenic DCs of Myd88 heterozygous or knockout mice that had been infected with F-MLV. As shown earlier (Figure 2), F-MLV infection caused up-regulation of CD80 on all three major splenic DC subsets in heterozygous mice. In the Myd88 knockout mice, the CD11c^+^ B220^−^ CD8α^−^ DCs exhibited little reduction in up-regulation of CD80 (Figure 6, left panel). CD11c^+^ B220^−^ CD8α^+^ DCs and CD11c^int^ B220^+^ CD8α^−^ DCs, by contrast, exhibited more pronounced reduction in maturation, but still exhibited some response to F-MLV (Figure 6, middle and right panels). This suggests that both Myd88-dependent and independent pathways contribute to the maturation of DCs in response to F-MLV, and that the contribution made by Myd88-dependent signaling varies somewhat between different DC subsets.

### CD8^+^ and CD4^+^ T cell responses to F-MLV are present but reduced in the absence of Myd88

**Figure 7:**
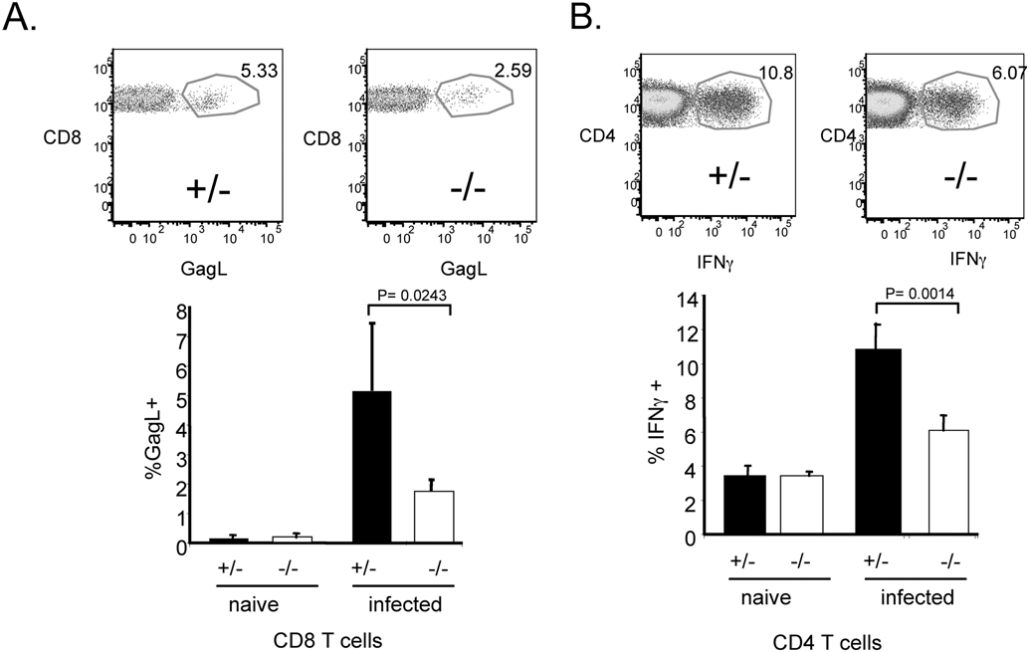
T cell responses to F-MLV are partially dependent on Myd88. Myd88 knockout mice and heterozygous littermates were infected with F-MLV for 14 days. (A) Splenocytes were stained with TCRβ, CD8α, and the D^b^GagL tetramer, and the proportion of TCRβ^+^ CD8^+^ cells that were GagL^+^ was measured. A representative FACS plot gated on TCRβ^+^ CD8^+^ cells is shown in the upper panel. For the bar graph in the lower panel, each bar is the average of four mice. (B) Splenocytes were stained for TCRβ, CD4, and intracellular interferon-γ. The proportion of TCRβ^+^ CD4^+^ T cells that expressed interferon-γ was measured. Each bar is the average of four mice. The data shown are representative of three independent experiments.

Previous studies have shown that immune control of F-MLV infection depends on all three of the major lymphocyte populations - CD4^+^ T cells, CD8^+^ T cells and B cells [24]. We wished to determine if T cell responses to F-MLV were defective in Myd88 knockout mice. We infected Myd88 heterozygous and homozygous mutant mice with F-MLV, and at 13 dpi analyzed CD8 T cells by staining with the D^b^-GagL tetramer (Figure 7A). In heterozygous mice, approximately 5–8% of the CD8^+^ T cell population stained positive with this tetramer (Figure 7A, left panel). In naïve mice, CD8 cells were not significantly stained (0.2%). In homozygous mutant mice, the proportion of CD8^+^ T cells that was D^b^-GagL^+^ was significantly reduced to approximately 2% (Figure 7A, right panel). This indicates that the CD8^+^ T cell response to F-MLV is partially dependent on Myd88 signaling, but that Myd88-independent mechanisms can compensate in its absence.

To determine if the CD4^+^ T cell response to F-MLV was affected by loss of Myd88, we analyzed CD4^+^ T cells in infected Myd88 heterozygous or homozygous mutant mice by intracellular staining for interferon-gamma (IFNγ) expression. In naïve heterozygous and knockout mice, roughly 3% of the CD4^+^ T cells stained positive for IFNγ (Figure 7B). In infected heterozygous mice, 11% of the CD4^+^ T cells were IFNγ positive, indicative of a robust Th1 response to F-MLV. By contrast, the proportion of IFNγ positive CD4 T cells in Myd88 knockout mice was reduced to 6%. These results indicate that both the CD8^+^ and CD4^+^ T cell responses to F-MLV are present but reduced in the absence of Myd88.

### Myd88 is required for the generation of neutralizing antibodies to F-MLV

**Figure 8:**
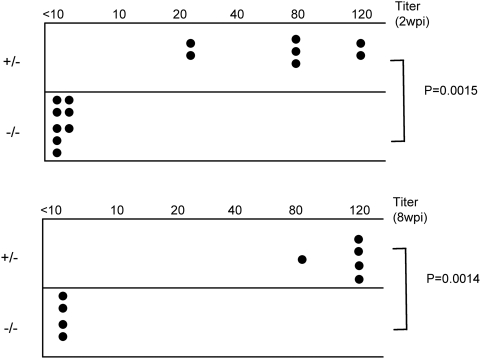
Myd88 is required for a neutralizing antibody response against F-MLV. Serum was isolated from the peripheral blood of F-MLV–infected *Myd88* ^−/−^ and *Myd88* ^+/−^ littermate controls at 2 wpi and 8 wpi. The titer of neutralizing antibodies in the serum was measured. Each dot represents an individual mouse. The data shown are from one of four independent experiments.

To determine whether the B cell/antibody response to F-MLV was affected by loss of Myd88, we measured the titer of F-MLV–neutralizing antibodies in the serum of infected Myd88 mutant mice. At 14 dpi, heterozygous control mice exhibited a strong neutralizing antibody response to F-MLV with a titer typically in the range of 80 (Figure 8, upper panel). By contrast, in the serum of Myd88-deficient mice, neutralizing antibodies were undetectable. We also measured neutralizing antibody titers at eight weeks post-infection (Figure 8, lower panel), and found strong antibody titers in the serum of heterozygous mice but undetectable F-MLV–neutralizing activity in the homozygous mutant animals. Since it is known that B cells and neutralizing antibodies are required to control F-MLV, the results suggest that the lack of a neutralizing antibody response is a significant contributor to the failure of Myd88-deficient mice to control F-MLV.

### F-MLV–specific IgG is absent from serum of infected mice lacking Myd88 or CD11c^+^ DCs

**Figure 9:**
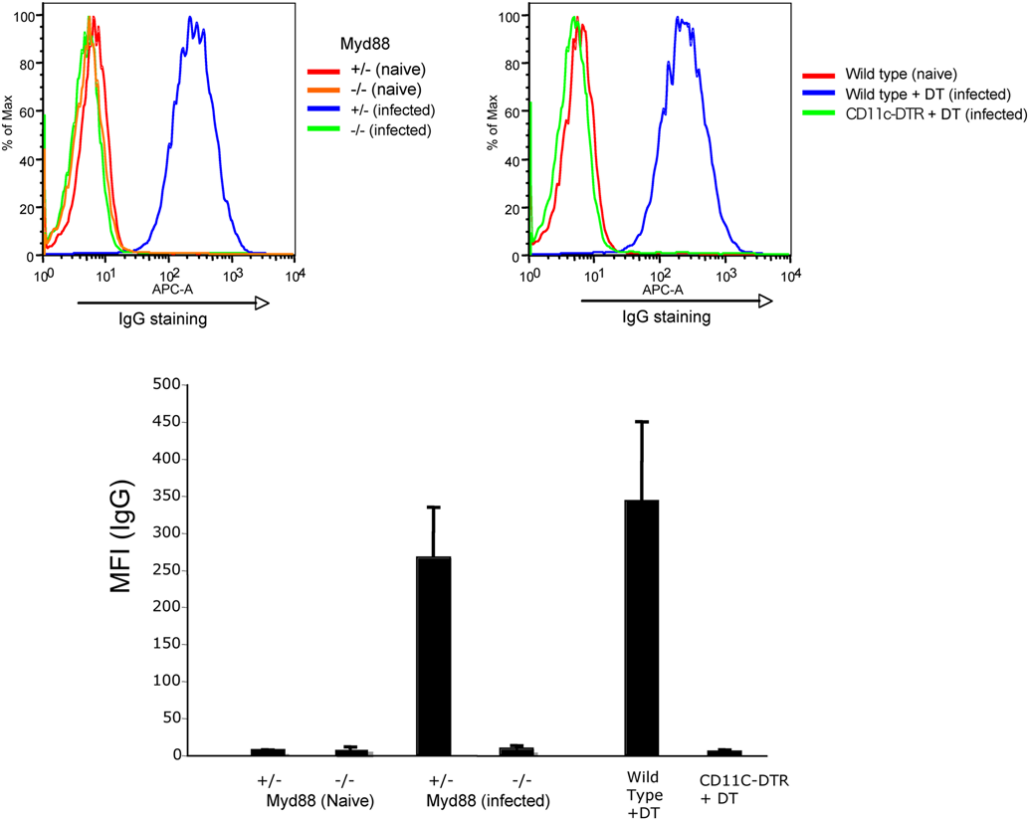
F-MLV–specific IgG is absent from serum of infected mice lacking Myd88 or CD11c^+^ DCs. Serum samples from naïve or infected mice were assayed for the presence of F-MLV–specific IgG, by incubation with a suspension of F-MLV–infected cells, followed by secondary staining for mouse IgG. The cells were then analyzed by flow cytometry. Representative stainings for Myd88 knockout mice (upper left) and CD11c^+^ DC-depleted mice (upper right) are shown. Mean fluorescence intensities (MFI) for the average of five mice were calculated for each condition and plotted (lower panel). The data shown are from one of two independent experiments.

The absence of a detectable neutralizing antibody titer in Myd88-deficient or CD11c^+^ DC-deleted mice could reflect a general loss of serum antibodies to F-MLV or a defect specifically in antibodies that neutralize infection. To distinguish between these possibilities, we measured F-MLV–specific IgG levels in the serum of these mice at 14 dpi. F-MLV–specific antibodies were detected by incubating serum samples from infected mice with a suspension of an F-MLV–infected cell line, followed by staining for mouse IgG and flow cytometry. Staining activity was present in the serum of infected wild-type or Myd88 heterozygous mice, but absent from the serum of naïve mice (Figure 9). Also, serum from wild-type or Myd88 heterozygous mice did not stain an uninfected cell line, indicating that the staining was specific for F-MLV (not shown). Significantly, F-MLV–specific IgG was completely absent from the serum of infected Myd88-deficient or DC-depleted mice. These data argue that Myd88 and CD11c^+^ DCs regulate the generation of total anti F-MLV IgG levels, and not only the amount of antibodies that neutralize viral infection.

Since B cells, CD4^+^ T cells, and DCs are all known to regulate antibody responses, it is possible that an increased level of viral infection of one of these cell types during the acute phase was responsible for the antibody defect in Myd88-deficient mice. To examine this possibility, we examined the proportion of each of these cells that was infected with F-MLV at 6 dpi by staining splenocytes of infected mice for cell surface expression of the viral GlycoGag protein. Neither B cells, CD4^+^ T cells, nor DCs exhibited significantly higher proportions of infected cells in Myd88 knockout mice at this timepoint (not shown).

### Serum from wild-type infected mice can rescue control of F-MLV in Myd88-deficient mice

**Figure 10:**
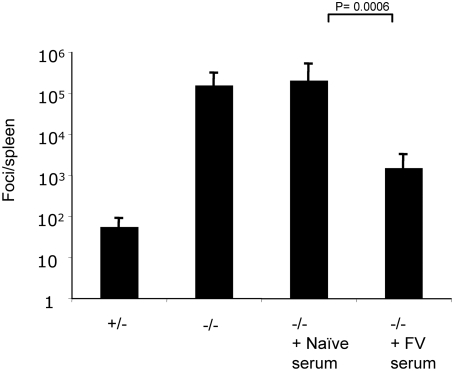
Transfer of serum from infected wild-type mice to Myd88 knockout mice rescues control of F-MLV. Myd88 knockout mice or heterozygous littermates were infected with F-MLV. At 7 dpi, infected mice were injected intraperitoneally with 0.5 ml serum from uninfected C57BL/6 mice or from C57BL/6 mice that had been infected with F-MLV for 14 days. At 14 dpi, the levels of viral focus-forming units in the spleens of infected mice were analyzed. Each bar represents the average of 4–7 mice from one of two independent experiments.

Because the most profound defect associated with the poor control of F-MLV infection in Myd88-deficient mice was the lack of a neutralizing antibody response, we asked whether passive transfer of immune serum from wild-type mice could rescue control of F-MLV in the mutant mice. We infected Myd88 heterozygous or homozygous mutant mice with F-MLV, and at 7 dpi homozygous mice were injected intraperitoneally with 0.5 ml of serum taken either from naïve C57BL/6 mice or from mice that had been infected with F-MLV 14 days earlier. Consistent with our previous observations, at 14 dpi heterozygous mice had low numbers of viral foci in their spleens, while Myd88-deficient mice exhibited approximately 1000-fold higher numbers of foci (Figure 10). Knockout mice that had received naïve serum had virus titers similar to untreated knockout mice. However, Myd88-deficient mice that had received serum from wild-type F-MLV–infected mice had virus levels that were more than 100-fold lower, with approximately 10^3^ foci per spleen. This indicates that serum from wild-type infected mice can rescue control of F-MLV in Myd88-deficient mice, and is consistent with the finding that the most important role of Myd88 in F-MLV infection is the generation of a virus-specific neutralizing antibody response.

## Discussion

Although innate immune detection of viral pathogens is considered to be important for the initiation of adaptive immune responses, for most viruses, including retroviruses, the specific sensing pathway has not been identified. Our results demonstrate a crucial role for CD11c^+^ DCs and Toll-like receptor signaling in the antibody response to retroviral infection. We have also identified a significant but less critical role for TLRs in CD4^+^ and CD8^+^ T cell responses to retroviral infection. Myd88-deficient mice sustain high titers of F-MLV for several months post-infection, indicating that the response is not simply delayed. To our knowledge, this is the first report of Myd88 or TLR signaling playing an essential role in immune control of a virus that is primarily mediated *via* the anti-viral antibody response. Similarly, previous studies of the effect of DC depletion in immune control of pathogens have focused on T cell responses, rather than on antibody-mediated control [23],[25]. Our results demonstrate that depletion of DCs leads to a loss of immune control of F-MLV, and this was found to coincide with a complete loss of F-MLV–specific IgG but no significant reduction in CD8^+^ T cells responses.

The role of TLRs in B cell dependent immune responses has been somewhat controversial. Pasare and Medzhitov found that Myd88 knockout mice were defective at generating antibodies specific for the model antigens OVA and HSA in the presence of LPS [26]. Furthermore, they found this defect to be at least partly B cell intrinsic, since mice lacking mature B cells due to a disruption of the Ig μ chain (μMT mice) that had received B cells lacking Myd88, but not mice that had received wild-type B cells, also had defective antibody responses. This view has been challenged by Gavin and coworkers [27] who found that *Myd88^−/−^;Trif^LPS2/LPS2^* mice were able to mount apparently normal antibody responses to the model antigen trinitrophenol-hemocyanin given in complete Freund's adjuvant. However, neither of these studies examined the requirement of TLR signaling for an antibody response in the context of a live viral infection, nor did they examine the ability of the antibody response to neutralize an infectious pathogen. The issue of whether or not TLR signaling is important for effective neutralizing antibody responses is of profound importance for vaccine design, and the work that we describe here strongly suggests that, for retroviral pathogens, functional TLR signaling is indeed required for the antibody response.

Some previous reports have indicated a role for TLRs in the development of antibody responses during viral infection. Heer and coworkers found that, in Myd88 knockout mice, antibodies specific for influenza exhibited defective class switching [28]. Furthermore, it has been shown that long term maintenance of antibodies to polyoma virus required Myd88, although Myd88-deficient mice were still able to control polyoma virus infection *in vivo* [29]. Interestingly, Myd88-deficient mice that were infected with Vesicular Stomatitis Virus exhibited higher levels of VSV-specific IgG1 but lower levels of IgG2a relative to wild type mice, while the neutralizing antibody titer was somewhat lower [30],[31].

Previous studies of the roles played by TLRs in anti-viral immune responses have shown significant differences from virus to virus. For example, Myd88 is required for the CD8^+^ T cell response to the arenavirus LCMV [32],[33], but not for the CD8^+^ T cell response to influenza [28]. The reason for the differential requirement for TLRs in the immune response to different viruses is not clear but could be related to the structural features of the virus, cell tropism, or the details of the replication strategy used by the virus.

The specific cell type(s) in which TLR signaling is required for the antibody response to F-MLV is not clear. TLR signaling in DCs promotes the expression of costimulatory molecules such as CD80 and CD86 that are important for triggering CD4^+^ and CD8^+^ T cell responses. CD4^+^ T cells, in turn, play an important role in activating B cells. Thus, the effect of DCs and TLR signaling on B cell responses could be mediated through CD4^+^ T cells. However, upregulation of CD80 on the DC population (CD11c+, B220−, CD8α−) that is known to control CD4^+^ T cell responses was only slightly reduced in the absence of Myd88. Similarly, the CD4^+^ T cell response to F-MLV was reduced but not absent in Myd88 knockout mice. DCs are known to activate B cells directly in a T cell independent manner by FcγRIIB-mediated recycling of whole antigen, but this process is not known to be TLR dependent [34]. It is possible that DCs release cytokines in a Myd88-dependent manner that directly affects B cell responses. Also, since B cells express TLRs [35], it is possible that TLR signaling is directly required in B cells to generate F-MLV–specific antibodies. Conditional deletion of Myd88 in DCs or B cells would address the relative contribution of Myd88-dependent signaling in these cell types.

These results also suggest that DC-independent and Myd88-independent pathways contribute to the development of the T cell responses. Since retroviral genomes fold into structures that contain dsRNA, it is possible that TLR3 and TRIF could be involved. Also, since the retroviral replication cycle involves the generation of dsDNA in the cytoplasm of infected cells, it its possible that a cytoplasmic DNA sensor such as DAI contributes to retroviral detection [36]. Cellular molecules that are released from damaged cells, such as uric acid and ATP, have also been shown to have the ability to stimulate immune responses [37], but most retroviruses, including F-MLV, are non-cytopathic and do not cause the death of infected cells.

Since DC-depleted mice can still mount a significant CD8^+^ T cell response to F-MLV, it is possible that another antigen presenting cell type is sufficient to mediate this function. Depletion of other antigen presenting cells such as B cells and macrophages could be used to address this issue, although B cell-deficient μMT mice are still able to mount a CD8^+^ T cell response to F-MLV, indicating that an essential role for B cells in the CD8 T cell response is unlikely [38].

Could manipulation of TLR signaling potentially be used to enhance effectiveness of an HIV vaccine? It has been argued that effective vaccines, such as live attenuated vaccines, are able to provide effective protection because they stimulate TLRs. Furthermore, most effective vaccines involve the use of adjuvants that contain TLR ligands, although for many of these the role of TLR signaling has not been conclusively demonstrated. One study found that administration of the TLR9 ligand CpG enhanced the cellular and humoral response induced by a Gag prime boost vaccine [39]. Also, it has been demonstrated that conjugating Gag to the TLR7 agonist R-848 enhanced Gag-specific Th1 and CD8 responses in mice [40]. It remains to be determined, however, whether either of these approaches will enhance protection from HIV infection.

B cell vaccines and T cell vaccines have by themselves been unsuccessful in eliciting protection from HIV infection. For F-MLV, Messer and coworkers found that neither vaccination with the cell line FBL3 that expresses the viral protein GlycoGag to stimulate T cell responses nor passive transfer of neutralizing antibodies is sufficient to elicit sterilizing immunity [38]. However, the combination of immune serum and the FBL3 T cell vaccine did result in sterilizing immunity.

The issue of why HIV infected patients fail to develop an effective neutralizing antibody response remains unresolved. If TLR signaling is involved in the antibody response to HIV, it is conceivable that viral disruption of TLR signaling could contribute to the failure of this response. However, virus specific antibodies are not absent in HIV infected persons, but they lack sufficient neutralizing activity to control the viremia [41]. Our findings indicate that Myd88 plays a fundamental role in the generation of a total retroviral antibody response rather than specifically neutralizing antibodies.

In summary, these results demonstrate a critical role for DCs and TLR signaling in the humoral immune response to retroviral infection, and provide the first *in vivo* examination of the role played by TLRs in immune control of retroviral pathogens. Understanding precisely how TLR signaling contributes to the anti-retroviral immune response will help to guide the development of vaccines for retroviral pathogens such as HIV.

## Materials and Methods

### Mice

Wild-type C57BL/6 mice were obtained from Taconic Farms. Myd88-deficient mice were a gift from R. Medzhitov and were backcrossed to the C57BL/6 background for 5 generations. The Myd88-deficient mice were originally developed in the laboratory of S. Akira (Osaka, Japan). For all experiments, littermate controls were used. CD11c-DTR mice have been previously described [23], and had been generated on C57BL/6 background. Ethical approval for mouse experiments was obtained from the NYU Medical Center Institutional Animal Care and Use Committee.

### Viral infections

F-MLV stocks were generated by infection of Balb/c mice with 5000 focus-forming units of F-MLV. At 14 dpi, a 10% spleen homogenate suspension was made and titered by focus-forming assay. For experiments, mice at 6–8 weeks of age were infected with 7000 focus-forming units of F-MLV by retro-orbital intravenous injection.

### Focus-forming assays

Splenocytes in suspension were serially diluted and plated on a subconfluent monolayer of the F-MLV–susceptible *Mus Dunni* cell line. For platings, 5×10^1^–5×10^6^ splenocytes were added to 2×10^5^ *Mus Dunni* cells in 6 well plates. After 3 days of co-culture in RPMI medium, the cells were fixed with methanol and stained for 2 hours with the monoclonal antibody mAb720, which recognizes the F-MLV envelope glycoprotein. The cells were washed three times with phosphate-buffered saline and stained with anti-mouse IgG1 horseradish peroxidase for one hour. Foci were then visualized by adding Amino ethyl Carbazole/Hydrogen peroxide substrate (Sigma).

### Bone marrow chimeras and CD11c^+^ DC depletions

Wild-type C57BL/6 mice at 6–8 weeks of age were lethally irradiated with two doses of 550 Rads separated by six hours and then injected intravenously with bone marrow cells from wild-type or CDllc-DTR transgenic mice. The chimeras were then kept for 6 weeks to permit engraftment of donor bone marrow. During this period, the mice were supplied with antibiotics in their drinking water to prevent infection. Depletion of CD11c^+^ dendritic cells was carried out by fifteen days of daily intraperitoneal injection of 200 ng of diphtheria toxin (Sigma).

### Intracellular cytokine staining

Splenocytes were plated in RPMI containing 50 ng/mL PMA and 1 uM ionomycin plus GolgiStop (BD Biosciences). After 3 h stimulation, cells were washed in PBS and stained for cell surface markers. The cells were then washed in PBS and resuspended in permeabilization-fixing buffer (BD Biosciences). After incubation at 4°C for 45 minutes, the cells were washed and resuspended in permeabilization/wash buffer containing anti-IFNγ-PE antibody. Staining was carried out at for 45–60 min at 4°C. Finally, the cells were washed in 1–2 mL BD perm/wash buffer and resuspended in PBS for analysis.

### Neutralizing antibody titers

To measure neutralizing antibody titers in infected mice, peripheral blood was obtained by retro-orbital bleed, and serum extracted by centrifugation. The serum was diluted in a two-fold dilution series, and mixed with an F-MLV sample before plating on *Mus Dunni* cells. Three days later, when the *Mus Dunni* cells were confluent, infected foci were visualized. The neutralizing antibody titer was defined as the maximum serum dilution that reduced the number of F-MLV foci by at least 75%.

### F-MLV–specific IgG assay

Serum samples from infected or naïve mice were diluted in phosphate buffer saline with 2% fetal bovine serum. Samples were then incubated with a suspension of the chronically F-MLV–infected cell line NRK/SFFV-57 on ice for 45 minutes to capture F-MLV–specific antibodies. 100,000 cells were used per staining. The cells were then washed twice and resuspended in PBS/FBS. Secondary staining using Allophycocyanin-conjugated anti-mouse IgG (eBiosciences) was performed, then the cells were analyzed by flow cytometry.

### Statistical analysis

To determine statistical significance, P values for specific experiments were calculated using the Student's T- test for Figures 3B, 4A, and 7, and by Mann-Whitney test for Figures 3A, 4B, 8, and 10.
